# Resistance Detection and Transmission Risk Analysis of Pig-Derived Pathogenic *Escherichia coli* in East China

**DOI:** 10.3389/fvets.2021.614651

**Published:** 2021-04-30

**Authors:** Xiaoting Li, Haibin Liu, Sai Cao, Ping Cheng, Fulei Li, Muhammad Ishfaq, Jichao Sun, Xiuying Zhang

**Affiliations:** ^1^Heilongjiang Key Laboratory for Animal Disease Control and Pharmaceutical Development, Northeast Agricultural University, Harbin, China; ^2^Department of Basic Veterinary Science, College of Veterinary Medicine, Northeast Agricultural University, Harbin, China

**Keywords:** ESBLs, *E. coli*, multi-drug resistance, resistance gene, resistance phenotype

## Abstract

**Objective:** Antibiotics play an essential role in the treatment and prevention of diseases in pig farms. However, the irrational use of antibiotics leads to the emergence of multi-drug resistance of bacteria, which poses a critical threat to the efficacy of antibiotic treatments. Therefore, the study is designed to analyze the drug resistance of pathogenic *Escherichia coli* isolated from large-scale pig farms in East China, which provides a theoretical basis for precisely targeted clinical drugs in swine farms.

**Method:** The pathogenic *E. coli* were isolated and identified from clinical samples of swine farms, and the drug resistance of pathogenic *E. coli* was detected by antimicrobial susceptibility test (AST) and minimum inhibitory concentration test (MIC). Moreover, the prevalence of plasmid-mediated β-lactam resistance genes was analyzed by PCR.

**Results:** A total of 67 pathogenic *E. coli* were isolated from 152 samples collected from 20 large-scale pig farms in East China. All isolated pathogenic *E. coli* are associated with severe drug resistance. Moreover, 70% of isolated pathogenic *E. coli* is resistant to more than four antibiotics. Besides, there were 19 serotypes including O2, O4, O5, O6, O14, O26, O38, O42, O49, O57, O92, O93, O95, O101, O121, O131, O143, O158, and O161, of which the O4 and O92 serotype were the main serotypes in swine farms. The main extended-spectrum beta-lactamases (ESBLs)-encoding genes in East China were *bla*_CTX−M_, *bla*_TEM_, and *bla*_OXA_ by the detection of the ESBLs encoding genes of porcine pathogenic *E. coli*. The conjugation assays showed that a total of 30 transconjugants were obtained by conjugation, which indicated that drug resistance genes could be transmitted horizontally through conjugative plasmids.

**Conclusion:** The isolated pathogenic *E. coli* were all multi-drug resistant, and especially O4 and O92 were the main serotypes. The β-lactam resistance genes were prevalent in large-scale pig farms in East China, which provided a theoretical basis for the prevention and control of pig-derived pathogenic *E. coli* in the future.

## Introduction

*Escherichia coli*, gram-negative bacteria, is one representative number of the genus *E. coli*. It is the leading microbial community in the intestinal tract of common animals, and its survival mode is mainly heterotrophic (1). *Escherichia coli* is an opportunistic pathogen. Livestock and poultry infected by pathogens often have local or systemic inflammatory symptoms, severe diarrhea, and even sepsis (2). Colibacillosis has become a stubborn disease in the modern breeding industry because of its high morbidity and mortality (3–5). Antibacterial drugs can effectively treat colibacillosis. However, overuse and misuse of broad-spectrum antibiotics accelerate the growing resistance and even emergence of a superbug (6–8).

Since the 1940s, antibiotics have transformed medicine, and most infectious diseases can be effectively controlled by the appropriate use of the correct drugs (9, 10). After that, a large number of natural β-lactam antibiotics were discovered. Also, a series of semi-artificial and synthetic β-lactam antibiotics have been obtained through the structural transformation of antibiotics. Penicillin and cephalosporins are the most common β-lactam antibiotics. In addition, there are some new types, such as cephalosporins, oxycephalosporins, monocyclic β-lactams, carbapenems and β-lactamase inhibitors, etc. (11–13). The mechanism of action of β-lactam drugs is relatively similar by inhibiting the production of mucopeptide synthase in the cell wall of the bacteria to prevent the production of the mucopeptides, and then achieve the effect of destroying the cell wall of the bacteria, and ultimately cause the death of the bacteria (14, 15). In addition, the autolysin enzyme activity of bacteria can also be stimulated by β-lactam drugs to achieve the purpose of inhibiting bacterial growth (16). However, bacterial resistance has gradually emerged with the large-scale use of these drugs (17–19).

These bacteria containing extended-spectrum beta-lactamases (ESBLs) are resistant to a variety of β-lactam drugs (20). ESBLs are mostly produced by Enterobacteriaceae bacteria, which are relatively sensitive to some β-lactamase inhibitors such as clavulanic acid, and are mainly derived from β-lactamase genes (21, 22). It is called extended-spectrum β-lactamase because of its complete hydrolysis substrate and strong drug resistance (23). In *K. pneumoniae*, the SHV-type ESBLs through the 1990s were successful and widely distributed (24). Currently, many types of plasmid-mediated ESBLs have been found and divided into five categories: TEM type, SHV type, CTX-M type, OXA type, and other types (25). ESBLs-producing *E. coli* strains have multi-drug resistance, mainly because the resistance genes encoding ESBLs are often associated with other resistance genes, such as aminoglycosides, chloramphenicol, fluoroquinolones, sulfonamides, and tetracyclines resistance genes, on the same plasmid (26). According to research, the transfer frequency of drug-resistant plasmids is about 10^−8^–10^−4^ (27). However, due to the widespread and common existence of *E. coli* and drug-resistant plasmids, the transfer and spread of drug resistance in *E. coli* are convenient and easy.

In this study, 20 large-scale pig farms were monitored in East China. The multi-drug resistance, drug resistance rate and drug resistance spectrum were analyzed, respectively. The research can provide scientific and sufficient theoretical basis for accurate and precise monitoring of *E. coli* drug resistance and the spread of drug resistance in the East China. The drug resistance analysis of pig-derived pathogenic *E. coli* can provide a scientific theoretical basis for rational drug use and prevention of *E. coli* diseases.

## Materials and Methods

### Sample Collection

Anal and nasal swabs were collected from a total of 152 sick pigs and stored in Eswabs (Copan, Brescia, Italy) from 20 large-scale pig farms in East China (Supplementary Data 1). Nasal swab and anal swab which were collected from the same pig were counted as one sample. *Escherichia coli* was isolated and identified using eosin methylene blue agar and MacConkey agar plates and biochemical identification methods, respectively. The protocols used during this study were approved by the Northeast Agricultural University Institutional Animal Care and Use Committee, and all the animal care and treatment methods complied with the standards described in the Laboratory Animal Management Regulations (revised 2016) of Heilongjiang Province, China.

### Detection the Pathogenicity of *E. coli*

The *E. coli* solution was diluted to 5 × 10^8^ CFU/mL with normal saline and was inoculated into abdominal cavity of Kunming mice (Vital River, Beijing, China) with 0.01 mL/g body weight. A total of 760 mice were randomly assigned to 152 groups and each group was inoculated with one *E. coli* isolates. Some mice died due to the infection caused by pathogenic *E. coli*. The heart blood and liver of dead mice were collected under aseptic conditions. *Escherichia coli* in the samples were separated and purified. In addition, they were identified by eosin-methylene blue medium. The isolates that meet the growth characteristics of *E. coli* were isolated. Finally, the isolates were subjected to gram staining, microscopic examination, and biochemical identification.

### Antimicrobial Susceptibility Test and Minimum Inhibitory Concentration Test

Multidrug resistance was defined as the resistant to three or more antimicrobial subclasses. The AST of pig-derived pathogenic *E. coli* isolates was carried out according to the Kirby Bauer method recommended by the World Health Organization (WHO) (28). The following antimicrobial agents were purchased from the Chinese Institute of Veterinary Drug Control (Beijing, China): Streptomycin (S), gentamicin (GM), amikacin (AN), enrofloxacin (ENR), ciprofloxacin (CIP), doxycycline (DOX), ceftriaxone (CRO), ceftiofur (CTF), florfenicol (FLR), chloramphenicol (C), amoxicillin (AMX), sulfamethoxazole (SXT), fosfomycin (FOS), and polymyxin B (PB). *Escherichia coli* ATCC 25922 was used as a reference strain. Inhibitory zone diameter of 14 antimicrobial agents is shown in Supplementary Data 2. Besides, detected the MIC of these isolates, and the results were judged based on National Committee for Clinical Laboratory Standards (NCCLS) (Supplementary Data 3) (29). The reference strain *E. coli* ATCC 25922 was used as quality control for MICs.

### Determination the Serotype of Pig-Derived Pathogenic *E. coli*

The O-serotypes of *E. coli* of the isolates were determined *via* a slide agglutination test following the manufacturer's 2016 instructions (Ningbo Tianrun Biotechnology Company, Ningbo, Republic of China).

### Detection of ESBLs-Encoding Genes of Pig-Derived Pathogenic *E. coli*

The DNA extraction kit (TaKaRa, Dalian, China) was used to extract the DNA of pig-derived pathogenic *E. coli*, and the primers of ESBLs-encoding genes were designed according to the ESBLs-encoding gene sequence published by GenBank and reference reports. Designed and synthesized primers of TEM type (GenBank: KM434126.1), SHV type (GenBank: EU376967.1), CTX-M type (GenBank: KM211691.1), and OXA type (GenBank: KX894452.1), respectively. The sequence of the primers is shown in Table 1. DNA sequencing using purified PCR products was provided by ABI PRISM 3730XL Analyzer (Applied Biosystems, Foster City, CA, United States) in Shanghai Sangon Biotech, Co., Ltd., China. The database similarity searches for nucleotide sequences performed using the BLAST tool at the National Center for Biotechnology Information (NCBI) website 1.

**Table 1:** Sequences of primers used for PCR.

**Target gene**	**Primer name**	**Primer sequence (5^**′**^-3^**′**^)**	**Length (bp)**
*bla*_CTX−M_	*bla*_CTX−M−F_	TTTGCGATGTGCAGTACCAGTAA	544
	*bla*_CTX−M−R_	CGATATCGTTGGTGGTGCCATA	
*bla*_TEM_	*bla*_TEM−F_	ATGAGTATTCAACATTTCCGTG	861
	*bla*_TEM−R_	TTACCAATGCTTAATCAGTGAG	
*bla*_SHV_	*bla*_SHV−F_	AGCCGCTTGAGCAAATTAAAC	713
	*bla*_SHV−R_	ATCCCGCAGATAAATCACCAC	
*bla*_OXA_	*bla*_OXA−F_	GGCACCAGATTCAACTTTCAAG	564
	*bla*_OXA−R_	GACCCCAAGTTTCCTGTAAGTG	

### Conjugation Assay

The transferability of the ESBLs-carrying plasmid among different *E. coli* strains was examined using *E. coli* strain J53 as the recipient (30). Briefly, the donor and recipient strains were mixed at a ratio of 1:3 on a microporous membrane and incubated for 16 h. Then, the mixed bacterial solution was inoculated on tryptone soy agar plate containing 4 μg/ml ceftriaxone and 200 μg/ml sodium azide to select transconjugants. Transconjugants were confirmed by PCR and sequencing (31).

## Results

### Isolation and Identification of Pig-derived *E. coli*

As shown in (Supplementary Data 4), all isolates can ferment glucose, and most isolates can ferment lactose and xylose, but only some isolates can ferment sucrose, oxidase, urease, and VP. In addition, all isolates were negative in the inositol test but positive in MR and indole tests. The results of biochemical identification showed that 78 isolates were consistent with the biochemical characteristics of *E. coli*. These 78 isolates were, respectively, inoculated into mice, and then 67 isolates were found to cause diarrhea 6 h, and death of mice 36 h after injection. These isolates conform to the growth characteristics of *E. coli*, so they can be judged as pathogenic *E. coli*.

### AST and MIC of Pig-derived Pathogenic *E. coli*

Through the AST, it was found that all isolates showed multi-drug resistance (Table 2). The results of the drug resistance spectrum showed that 67 pig-derived pathogenic *E. coli* isolates showed multi-drug resistance. These isolates mainly showed resistance ranged from 4 to 11 drugs, of which 8 drug-resistant isolates accounted for 28.36%. The second most is resistant to 7 drugs, reaching 16.42%. Furthermore, 71.64% (48/67) showed resistance to 7 or more drugs.

**Table 2:** The resistant phenotype of 67 pathogenic *E. coli* isolates.

**Multiple antibiotic resistance**	**Resistant phenotype**	**Strain no**.
4	GM-CRO-FLR-SXT	H10
	S-GM-CRO-SXT	H33
	GM-CRO-CTF-SXT	H56
5	S-CRO-C-AMX/CA-SXT	H28
	GM-AN-CRO-CTF-SXT	H44
	S-GM-AN-CRO-CTF	H54
	GM-AN-CRO-FLR-SXT	H63
	S-GM-AN-CRO-AMX/CA	H64
	S-GM-AN-CRO-SXT	H47/H66
6	GM-AN-CRO-CTF-AMX/CA-SXT	H18
	GM-AN-ENR-CRO-FLR-C	H24
	GM-AN-CRO-FLR-C-SXT	H32
	S-GM-AN-CRO-AMX/CA-SXT	H35
	S-ENR-CIP-CRO-FLR-AMX/CA	H36
	GM-AN-CRO-CTF-C-SXT	H40
	S-GM-AN-CRO-C-SXT	H46
	S-GM-AN-CRO-FLR-SXT	H75/H78
7	S-GM-CRO-CTF-FLR-C-SXT	H2
	S-GM-AN-CRO-CTF-FLR-SXT	H6
	S-GM-AN-CRO-FLR-C-AMX/CA	H9
	S-GM-AN-CRO-FLR-C-SXT	H15/H51
	S-GM-CRO-FLR-C-AMX/CA-SXT	H73/H74
	S-GM-AN-CRO-C-AMX/CA-SXT	H19/H29/H48/H67
8	S-GM-AN-ENR-CRO-FLR-C-SXT	H21
	S-GM-AN-ENR-CIP-CRO-FLR-SXT	H45
	GM-AN-ENR-CRO-FLR-C-AMX/CA-SXT	H49
	S-GM-AN-CRO-FLR-C-SXT-FOS	H77
	S-GM-AN-CRO-CTF-C-AMX/CA-SXT	H37/H41/H42
	S-GM-AN-CRO-CTF-FLR-C-SXT	H30/H34/H43/H57
	S-GM-AN-CRO-FLR-C-AMX/CA-SXT	H20/H52/H55/H59
		H62/H65/H68/H69
9	S-GM-AN-ENR-CIP-CRO-FLR-SXT-FOS	H4
	GM-AN-ENR-CRO-CTF-FLR-C-AMX/CA-SXT	H27
	GM-AN-ENR-CIP-CRO-CTF-FLR-C-SXT	H31
	S-GM-AN-ENR-CRO-CTF-FLR-C-SXT	H53
	S-GM-AN-ENR-CIP-CRO-FLR-C-SXT	H61
	S-GM-AN-ENR-CRO-FLR-C-AMX/CA-SXT	H14/H25/H26
	S-GM-AN-CRO-CTF-FLR-C-AMX/CA-SXT	H38/H50
10	S-GM-AN-ENR-CIP-CRO-CTF-FLR-C-SXT	H1
	S-GM-AN-ENR-CRO-CTF-FLR-C-AMX/CA-SXT	H22
	S-GM-AN-ENR-CIP-CRO-FLR-C-AMX/CA-SXT	H3/H5/H17/H58/H60
11	S-GM-AN-ENR-CIP-CRO-CTF-FLR-C-AMX/CA-SXT	H11

As showed in the Table 3, the resistance rate test results showed that 67 *E. coli* isolates are completely resistant to CRO, and all isolates were susceptible to DOX and PB. The results of MIC are consistent with the AST, which further confirms the drug resistance phenotype and drug resistance rate of each strain. The above results indicate that the drug resistance of pathogenic *E. coli* from pigs in East China is relatively serious.

**Table 3:** The resistance rate of 67 pathogenic *E. coli* isolates to 14 antibiotics.

**Antibacterial drugs**	**Number of resistant isolates**	**Resistance rate**	**Number of intermediates isolates**	**Intermediate rate**	**Number of susceptible isolates**	**Susceptible rate**
S	56	83.58%	4	5.97%	7	10.45%
GM	65	97.02%	1	1.49%	1	1.49%
AN	59	88.06%	5	7.46%	3	4.48%
ENR	21	31.34%	37	55.22%	9	13.44%
CIP	12	17.91%	8	11.94%	47	70.15%
DOX	0	0	0	0	67	100%
CRO	67	100%	0	0	0	0
CTF	22	32.83%	7	10.45%	38	56.72%
FLR	48	71.64%	3	4.48%	16	23.88%
C	50	74.63%	8	11.94%	9	13.43%
AMX/CA	37	55.22%	12	17.91%	18	26.87%
SXT	62	92.54%	1	1.49%	4	5.97%
FOS	2	2.98%	0	0	65	97.02%
PB	0	0	0	0	67	100%

### The Serotype of Pig-Derived Pathogenic *E. coli*

**Figure 1:**
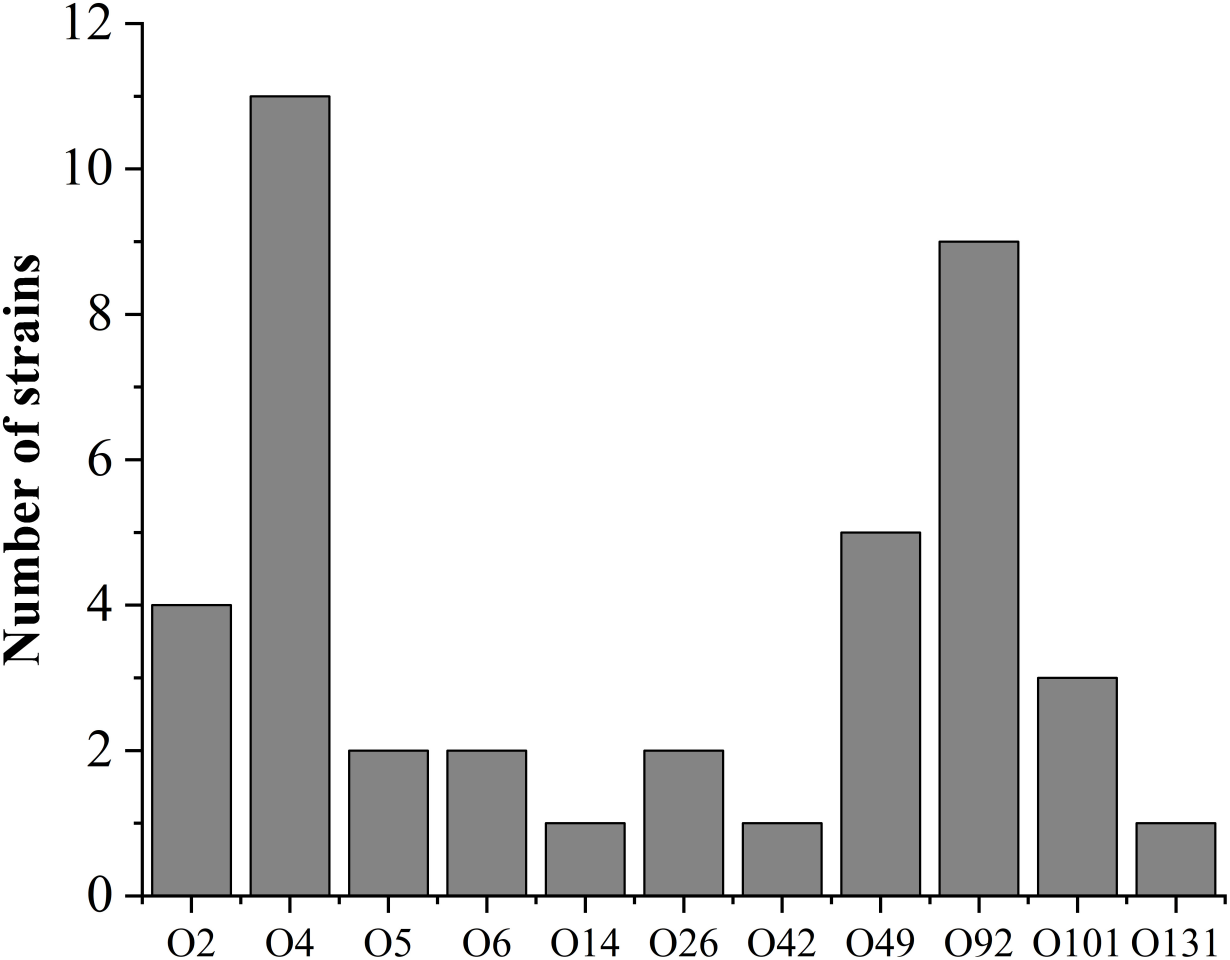
Results of serotype detection of pathogenic *E. coli* isolates.

The serotypes of pig-derived pathogenic *E. coli* were identified. The results showed that the others were successfully serotyped except for 4 isolates, accounting for 94.03% (63/67) of all pathogenic isolates. The serotype distribution is shown in Figure 1. There are 19 serotypes in 63 isolates, of which O4 and O92 are the main serotypes. Furthermore, some isolates were determined as mixed serotypes. All isolates of mixed serotypes were re-purified, and then the slide agglutination test was repeated. According to Table 4, the 34.92% (22/63) pathogenic isolates were mixed serotype.

**Table 4:** The number of mixed serotypes in pathogenic *E. coli* isolates.

**Serotype**	**Number of isolates**
O2, O121	1
O4, O26	4
O4, O92	1
O4, O93	1
O14, O121	1
O26, O158	1
O38, O57	2
O92, O93	1
O4, O14, O158	1
O14, O92, O161	2
O92, O95, O143	2
O93, O143, O161	1
O4, O92, O93, O143	4

### Detection of ESBLs-Encoding Genes of Pig-Derived Pathogenic *E. coli*

The detection rates of different ESBLs-encoding genes of 67 pathogenic *E. coli* isolates are shown in Table 5. The ESBLs-encoding genes with the highest detection rate were *bla*_CTX−M_ (70.15%), followed by *bla*_TEM_ (61.19%) and *bla*_OXA_ (25.37%), and *bla*_SHV_ were not detected.

**Table 5:** The detection rate of ESBLs-encoding genes of 67 pathogenic *E. coli* isolates.

**Target gene**	**Fragment size (bp)**	**Number of isolates**	**Percentage (%)**
*bla*_CTX−M_	544	47	70.15
*bla*_TEM_	861	41	61.19
*bla*_OXA_	564	17	25.37
*bla*_SHV_	713	0	0

### Transferability of ESBLs Genes

As shown in Table 6, the conjugation assay results showed that 30 isolates successfully conjugated, and the success rate of conjugation was as high as 44.78%, all 30 transconjugants showed 4 or more kinds of drug resistance. The resistance phenotypes of DOX and PB were not detected in the transconjugants, but the resistance phenotypes of the other 12 antibiotics were detected. In addition, the drug-resistant phenotypes of 7 transconjugants were completely consistent with those of the donor isolates. According to Table 7, the transfer rate of resistant phenotype to CIP, CTF and FOS reached 100%. One transconjugant showed a new drug-resistant phenotype, and the probability of mutation of the drug-resistant phenotype was 3.3%.

**Table 6:** The resistance phenotype of donor isolates and transconjugants.

**Strain number**	**Donor strain**	**Transconjugants**
H1	S-GM-AN-ENR-CIP-CRO-CTF-FLR-C-SXT	S-GM-AN-CIP-CRO-FLR-C
H6	S-GM-AN-CRO-CTF-FLR-SXT	S-GM-CRO-CTF-FLR-SXT
H9	S-GM-AN- CRO-FLR-C-AMX/CA	S-GM- CRO-FLR-C
H10	GM-CRO-FLR-SXT	GM-CRO-FLR-SXT
H15	S-GM-AN-CRO-FLR-C-SXT	S-GM-AN-FLR- SXT
H17	S-GM-AN-ENR-CIP-CRO-FLR-C-AMX/CA-SXT	AN-ENR-CIP-CROC-AMX/CA-SXT
H18	GM-AN- CRO-CTF- AMX/CA-SXT	CRO-CTF- AMX/CA-SXT
H19	S-GM-AN-CRO- C-AMX/CA-SXT	S-GM-AN- AMX/CA-SXT
H22	S-GM-AN-ENR-CRO-CTF-FLR-C-AMX/CA-SXT	S -ENR-CRO-CTF-FLR-C-AMX/CA-SXT
H24	GM-AN-ENR-CRO-FLR-C	GM-AN-ENR-CRO-FLR-C
H25	S-GM-AN-ENR-CRO-FLR-C-AMX/CA-SXT	S- AN-ENR-CRO-FLR-C-SXT
H27	GM-AN-ENR-CRO-CTF-FLR-C-AMX/CA-SXT	GM- ENR-CRO-CTF-FLR-SXT
H28	S-CRO- C-AMX/CA-SXT	S-CRO- C-AMX/CA
H30	S-GM-AN- CRO-CTF-FLR-C-SXT	S-GM-AN- CRO-CTF-FLR-C-SXT
H31	S-GM-AN- CRO-CTF-FLR-C-SXT	S- AN- CTF-FLR-C-SXT
H32	GM-AN-CRO-FLR-C-SXT	GM-AN-CRO-FLR- SXT
H40	GM-AN-CRO-CTF-C-SXT	AN-CRO-CTF-C-SXT
H43	S-GM-AN- CRO-CTF-FLR-C- SXT	S- AN- CRO-CTF-FLR-C
H45	S-GM-AN- ENR-CIP-CRO-FLR- SXT	S-GM-AN- ENR-CIP-CRO-FLR- SXT
H50	S-GM-AN- CRO-CTF-FLR-C- AMX/CA-SXT	GM-AN- CRO-CTF-FLR-C- SXT
H52	S-GM-AN- CRO-FLR-C- AMX/CA-SXT	S- AN-FLR-C- AMX/CA-SXT
H56	GM-CRO-CTF-SXT	GM-CRO-CTF-SXT
H59	S-GM-AN- CRO-FLR-C- AMX/CA-SXT	S-GM-AN- CRO-FLR-C-SXT
H61	S-GM-AN- ENR-CIP-CRO-FLR-C- SXT	S- ENR-CIP- CRO-FLR-C- SXT
H66	S-GM-AN- CRO-SXT	S-AN- C-SXT
H68	S-GM-AN- CRO-FLR-C-AMX/CA-SXT	CRO-FLR-C-AMX/CA-SXT
H73	S-GM- CRO-FLR-C-AMX/CA-SXT	S-GM- CRO-FLR-C-AMX/CA-SXT
H74	S-GM- CRO-FLR-C-AMX/CA-SXT	S-GM- CRO-FLR-C
H77	S-GM-AN- CRO-FLR-C-SXT-FOS	S-GM-AN- CRO-SXT-FOS
H78	S-GM-AN- CRO-FLR-SXT	S-GM-AN- CRO-FLR-SXT

**Table 7:** The resistance rate of donor isolates and transconjugants to 14 antibiotics.

**Antibacterial drugs**	**Donor strain**		**Transconjugants**		**Transfer rate (%)**
	**Number of resistant strains**	**Drug resistance rate (%)**	**Number of resistant strains**	**Drug resistance rate (%)**	
S	23	76.67	20	66.67	86.96
GM	29	96.67	18	60	62.07
AN	25	83.33	18	60	72
ENR	8	26.67	7	23.33	87.5
CIP	4	13.33	4	13.33	100
DOX	0	0	0	0	0
CRO	30	100	25	83.33	83.33
CTF	10	33.33	10	33.33	100
FLR	24	80	22	73.33	91.67
C	23	76.67	19	63.33	82.61
AMX/CA	14	46.67	8	26.67	57.14
SXT	28	93.33	24	80	85.71
FOS	1	3.33	1	3.33	100
PB	0	0	0	0	0

According to Tables 8, 9, the detection rate of *bla*_CTX−M_ was the highest among the 30 donor strains, and the detection rates of *bla*_TEM_ and *bla*_OXA_ were 43.33 and 26.67%, respectively. Among the 30 transconjugants, the detection rates of *bla*_CTX−M_, *bla*_TEM_, and *bla*_OXA_ were 23.33, 23.33, and 6.67%, respectively. Comparing the donor and the transconjugants, the transfer rates of the three-drug resistance genes *bla*_CTX−M_, *bla*_TEM_, and *bla*_OXA_ were 43.75, 53.85, and 25%, respectively.

**Table 8:** ESBLs-encoding genes carried by donor isolates and transconjugants.

**Strain**	**Drug resistance gene**	
	**Donor strain**	**Transconjugants**
H1	*bla*_CTX−M_, *bla*_OXA_	*bla*_CTX−M_
H6	*bla*_TEM_	*bla*_TEM_
H9	*bla*_CTX−M_	-
H10	*bla*_TEM_	-
H15	*bla*_CTX−M_	-
H17	*bla*_OXA_	-
H18	*bla*_CTX−M_	*bla*_CTX−M_
H19	*bla*_CTX−M_	-
H22	*bla*_TEM_	*bla*_TEM_
H24	*bla*_CTX−M_, *bla*_TEM_	*bla*_CTX−M_
H25	*bla*_TEM_, *bla*_OXA_	*bla*_OXA_
H27	*bla*_TEM_	-
H28	*bla*_CTX−M_	*bla*_CTX−M_
H30	*bla*_TEM_	*bla*_TEM_
H31	*bla*_CTX−M_	-
H32	*bla*_OXA_	*bla*O_XA_
H40	*bla*_CTX−M_	-
H43	*bla*_CTX−M_, *bla*_TEM_	*bla*_TEM_
H45	*bla*_CTX−M_	-
H50	*bla*_OXA_	-
H52	*bla*_TEM_	-
H56	*bla*_CTX−M_	*bla*_CTX−M_
H59	*bla*_OXA_	-
H61	*bla*_TEM_	-
H66	*bla*_TEM_	*bla*_TEM_
H68	*bla*_CTX−M_, *bla*_OXA_	*bla*_CTX−M_
H73	*bla*_CTX−M_, *bla*_TEM_	*bla*_TEM_
H74	*bla*_CTX−M_	-
H77	*bla*_TEM_, *bla*_OXA_	*bla*_TEM_
H78	*bla*_CTX−M_	*bla*_CTX−M_

**Table 9:** ESBLs-encoding genes of donor isolates and transconjugants.

**Target gene**	**Donor strain**		**Transconjugants**		**Transfer rate (%)**
	**Number of strains**	**Positive rate (%)**	**Number of strains**	**Positive rate (%)**	
*bla*_CTX−M_	16	53.33	7	23.33	43.75
*bla*_TEM_	13	43.33	7	23.33	53.85
*bla*_OXA_	8	26.67	2	6.67	25

## Discussion

Antibiotics are the foundation of modern medicine, and their use reduces mortality and prolongs life expectancy (32). However, Antibiotic-resistant bacteria that are difficult or impossible to treat are becoming increasingly common and are causing a global health crisis (33, 34). In this study, identified the pathogenic *E. coli* isolated from large-scale pig farms in East China, and analyzed its drug resistance to 14 commonly used antibiotics. The proportion of multi-drug resistant isolates accounted for 100% of the resistant isolates, and these isolates mainly showed resistance to varied from 4 to 11 drugs. These data all indicate that the drug resistance of *E. coli* in East China is mainly manifested as multidrug resistance. Accordingly, the phenomenon of drug resistance has been very serious.

CRO, GM, SXT, AN, S, C, FLR, and AMX/CA resistance rates have reached more than 50%, which may be due to the relatively common clinical use of these antibiotics (35–37). We found that 2.98% of the isolates were resistant to FOS, and all isolates were susceptible to DOX and PB. This is also due to the late availability of these drugs and the relatively small application range (38–40). These drugs might play a vital role in the prevention and treatment of multi-drug resistant *E. coli* in the future.

*Escherichia coli* is an opportunistic pathogen with many serotypes and large variations. At present, in pathogenic *E. coli*, the antigen structure of pathogenic bacteria is mainly divided into four types: O, K, H, and F antigens type, of which O antigen is the most common type (41). The serotypes of animal-derived *E. coli* prevalent in various regions are quite different, and the advantages of different pathogenic *E. coli* O antigen serotypes are different (42). There are currently 196 types of known *E. coli* O antigens, and the serotypes are divided into O1 to O187 types. Most of the genes related to the synthesis of O antigen in *E. coli* are arranged tightly on the chromosome, thus forming an O antigen gene cluster (43).

The serotype identification results showed that a total of 19 serotypes were included in these isolates, 34.92% (22/67) isolates had two or more serotypes, indicating the existence of O antigen cross-reaction. Serotypes are related to the pathogenicity of *E. coli*. Moreover, the predominant serotypes of pathogenic *E. coli* were different in different areas. Due to the weak antigen cross-protection between different serotypes, it is impossible to prepare an extended-spectrum vaccine that can cover all serotypes. This phenomenon will increase the difficulty of prevention of *E. coli* disease to a certain extent. In addition, the results showed that there was no direct correlation between serotype and genotype of drug resistance gene. Therefore, the diversity, variability and complexity of serotypes have become the factors that need to be carefully considered in the prevention of *E. coli* disease.

The transconjugants test results showed that 30 of the 67 pathogenic *E. coli* isolates were successfully conjugated, with a success rate of 44.78%. In addition, the transconjugants also showed multi-drug resistance, and the drug-resistant phenotype was reduced compared with the donor strains. However, compared with the donor isolates, the transconjugants showed a tendency that its resistance rate to 14 kinds of antibacterial drugs was exactly the same as that of the donor isolates. The above phenomenon shows that the resistance genes of *E. coli* located in the plasmid were transferred to the recipient isolates along with the plasmid, and therefore it showed resistance to antibiotics. Moreover, according to the statistical results of the drug-resistant phenotype transfer rate of donor isolates, drug-resistant genes can be horizontally transmitted and expressed with plasmids. In the experiment, one transconjugant showed a new drug-resistant phenotype. Compared with the donor isolates, it had a chloramphenicol-resistant phenotype. The reason for this phenomenon may be the result of gene mutation.

The production of ESBLs in Enterobacteriaceae is a well-known mechanism for resistance to antibiotics. In China, increasing studies reported the increasing detection rates of ESBLs-producing *E. coli* collected from infected patients in the clinic, and CTX-M types were identified to be the most common ESBLs in *E. coli* (44, 45). We found that transconjugants have transferred resistance genes, and 9 of them have the same resistance genotype as the donor isolates. In addition, among the 30 transconjugants, 7 recipient strains had TEM genes,7 recipient strains had CTX-M genes, and 2 recipient strains amplified OXA genes. This phenomenon should be attributed to the fact that the resistance genes of the donor strain were carried on the conjugative plasmid, and the resistance genes were transferred to the transconjugants with the conjugation of donor and recipient bacteria.

All findings suggested that these drug resistance genes of *E. coli* were located on the conjugate plasmid and can be transmitted and expressed horizontally in the plasmid. Among the various routes for the spread of drug-resistant genes, horizontal gene transfer by plasmid-mediated conjugation is regarded as one of the primary mechanisms. Through conjugation, DNA can be transferred by mobile plasmids between genera, phyla, and even significant domains. For example, *E. coli* shows resistance after obtaining exogenous drug-resistant genes. Conjugative plasmids of *E. coli* can be transmitted and diffused among the same species or different species, which leads to the spread of antibiotic resistance. The horizontal transmission of genes is directly affected by the success rate of conjugation between strains. In this study, the high conjugation rate and the high transfer rate of drug-resistant genes in this study also confirmed the fact that the drug-resistant transmission of pig-derived pathogenic *E. coli* in East China is relatively severe.

## Conclusion

The result of this study showed that 67 pathogenic *E. coli* isolates showed multidrug resistance, which indicated that the drug resistance of *E. coli* in East China was severe. Besides, the results showed that there was no direct correlation between serotype and genotype of drug resistance gene. Therefore, the diversity, variability and complexity of serotypes have become factors that need to be carefully considered in the prevention of *E. coli* disease. Finally, by analyzing the transfer of ESBLs-encoding genes between donor and recipient isolates, it was found that antibiotic resistance of porcine pathogenic *E. coli* could be transmitted to recipient isolates through conjugation.

## Data Availability Statement

The original contributions presented in the study are included in the article/Supplementary Material, further inquiries can be directed to the corresponding author.

## Ethics Statement

The animal study was reviewed and approved by Northeast Agricultural University Institutional Animal Care and Use Committee. Laboratory Animal Management Regulations (Revised 2016) of Heilongjiang Province, China.

## Author Contributions

XL conceived and designed the experiments and wrote the manuscript. HL, SC, PC, FL, and JS provided assistance of the experiments and collected samples. MI revised the manuscript. XZ supervised the experiments and revised the manuscript. All authors contributed to the article and approved the submitted version.

## Conflict of Interest

The authors declare that the research was conducted in the absence of any commercial or financial relationships that could be construed as a potential conflict of interest.
