# Supplementary material for: Resistance Detection and Transmission Risk Analysis of Pig-Derived Pathogenic Escherichia coli in East China
Source: Front Vet Sci. 2021 Apr 30;8:614651. doi: 10.3389/fvets.2021.614651 (PMC8119771; doi:10.3389/fvets.2021.614651)
Supplement: Supplementary file 1 [file Data_Sheet_1.docx]

**Supplementary Data**

Supplementary Data 1 Details of sample collection

| Province | number of farms | number of samples | average number |
| --- | --- | --- | --- |
| An Hui | AH-1 | 8 | 7 |
|  | AH-2 | 6 |  |
|  | AH-3 | 7 |  |
|  | AH-4 | 7 |  |
| Jiang Su | JS-1 | 6 | 9 |
|  | JS-2 | 11 |  |
|  | JS-3 | 9 |  |
|  | JS-4 | 10 |  |
| Shan Dong | SD-1 | 8 | 8 |
|  | SD-2 | 7 |  |
|  | SD-3 | 9 |  |
|  | SD-4 | 8 |  |
| Jiang Xi | JX-1 | 9 | 8 |
|  | JX-2 | 8 |  |
|  | JX-3 | 7 |  |
|  | JX-4 | 8 |  |
| Zhe Jiang | ZJ-1 | 6 | 7 |
|  | ZJ-2 | 11 |  |
|  | ZJ-3  ZJ-4 | 5  6 |  |

Supplementary Data 2. Inhibitory zone diameter of 14 antimicrobial agents

| Antimicrobial Agent | Drug content in drug sensitive paper (μg/ tablet) | Inhibitory zone diameter (mm) | | |
| --- | --- | --- | --- | --- |
|  |  | Resistant | Intermediate | Susceptible |
| S | 10 | ≥15 | 12-14 | ≤11 |
| GM | 10 | ≥15 | 13-14 | ≤12 |
| AN | 30 | ≥17 | 15-16 | ≤14 |
| ENR | 5 | ≥23 | 17-22 | ≤16 |
| CIP | 5 | ≥21 | 16-20 | ≤15 |
| DOX | 30 | ≥14 | 11-13 | ≤10 |
| CRO | 30 | ≥23 | 20-22 | ≤19 |
| CTF | 30 | ≥23 | 15-22 | ≤14 |
| FLR | 30 | ≥19 | 15-18 | ≤14 |
| C | 30 | ≥18 | 13-17 | ≤12 |
| AMX | 20 | ≥18 | 14-17 | ≤13 |
| SXT | 23.75 | ≥16 | 11-15 | ≤10 |
| FOS | 200 | ≥16 | 13-15 | ≤12 |
| PB | 300IU | ≥12 | 9-11 | ≤8 |

Supplementary Data 3. Susceptibility breakpoints of antimicrobial agents

| Antimicrobial Agent | MIC(μg/ml) | | |
| --- | --- | --- | --- |
|  | Susceptible | Intermediate | Resistant |
| S | ≤16 | 32 | ≥64 |
| GM | ≤4 | 8 | ≥16 |
| AN | ≤16 | 32 | ≥64 |
| ENR | ≤0.25 | 0.5-1 | ≥2 |
| CIP | ≤1 | 2 | ≥4 |
| DOX | ≤4 | 8 | ≥16 |
| CRO | ≤1 | 2 | ≥4 |
| CTF | ≤1 | 2 | ≥4 |
| FLR | ≤2 | 4 | ≥8 |
| C | ≤8 | 16 | ≥32 |
| AMX | ≤4 | 8 | ≥16 |
| SXT | ≤38 | - | ≥76 |
| FOS | ≤64 | 128 | ≥256 |
| PB | ≤64 | 128 | ≥256 |

Supplementary Data 4. Biochemistry reaction result of 78 isolated strains

| Strain | Lactose | Oxidase | Glucose | Urease | MR | Sucrose | Indol | Xylose | VP | Inositol |
| --- | --- | --- | --- | --- | --- | --- | --- | --- | --- | --- |
| H1 | ＋ | － | ＋ | － | ＋ | － | ＋ | ＋ | － | － |
| H2 | ＋ | － | ＋ | － | ＋ | － | ＋ | ＋ | － | － |
| H3 | ＋ | － | ＋ | － | ＋ | － | ＋ | ＋ | － | － |
| H4 | ＋ | － | ＋ | － | ＋ | － | ＋ | ＋ | － | － |
| H5 | ＋ | － | ＋ | － | ＋ | － | ＋ | ＋ | － | － |
| H6 | ＋ | － | ＋ | － | ＋ | ＋ | ＋ | ＋ | － | － |
| H7 | ＋ | － | ＋ | － | ＋ | － | ＋ | ＋ | － | － |
| H8 | ＋ | － | ＋ | － | ＋ | － | ＋ | － | － | － |
| H9 | ＋ | － | ＋ | － | ＋ | － | ＋ | ＋ | － | － |
| H10 | ＋ | － | ＋ | － | ＋ | － | ＋ | ＋ | － | － |
| H11 | ＋ | － | ＋ | － | ＋ | － | ＋ | ＋ | － | － |
| H12 | ＋ | － | ＋ | － | ＋ | － | ＋ | ＋ | － | － |
| H13 | ＋ | － | ＋ | － | ＋ | － | ＋ | － | － | － |
| H14 | － | － | ＋ | － | ＋ | － | ＋ | ＋ | － | － |
| H15 | ＋ | － | ＋ | － | ＋ | － | ＋ | ＋ | － | － |
| H16 | ＋ | － | ＋ | － | ＋ | － | ＋ | ＋ | － | － |
| H17 | ＋ | － | ＋ | － | ＋ | － | ＋ | ＋ | － | － |
| H18 | ＋ | － | ＋ | － | ＋ | ＋ | ＋ | ＋ | － | － |
| H19 | ＋ | － | ＋ | － | ＋ | － | ＋ | ＋ | － | － |
| H20 | ＋ | － | ＋ | － | ＋ | － | ＋ | － | － | － |
| H21 | ＋ | － | ＋ | － | ＋ | － | ＋ | ＋ | － | － |
| H22 | ＋ | － | ＋ | － | ＋ | － | ＋ | ＋ | － | － |
| H23 | ＋ | － | ＋ | － | ＋ | － | ＋ | ＋ | － | － |
| H24 | ＋ | － | ＋ | － | ＋ | － | ＋ | ＋ | － | － |
| H25 | ＋ | － | ＋ | － | ＋ | － | ＋ | ＋ | － | － |
| H26 | ＋ | － | ＋ | － | ＋ | － | ＋ | ＋ | － | － |
| H27 | ＋ | － | ＋ | － | ＋ | － | ＋ | ＋ | － | － |
| H28 | － | － | ＋ | － | ＋ | － | ＋ | ＋ | － | － |
| H29 | ＋ | － | ＋ | － | ＋ | － | ＋ | ＋ | － | － |
| H30 | ＋ | － | ＋ | － | ＋ | － | ＋ | － | － | － |
| H31 | ＋ | － | ＋ | － | ＋ | － | ＋ | ＋ | － | － |
| H32 | ＋ | － | ＋ | － | ＋ | ＋ | ＋ | ＋ | － | － |
| H33 | ＋ | － | ＋ | － | ＋ | － | ＋ | ＋ | － | － |
| H34 | － | － | ＋ | － | ＋ | － | ＋ | ＋ | － | － |
| H35 | ＋ | － | ＋ | － | ＋ | － | ＋ | ＋ | － | － |
| H36 | ＋ | － | ＋ | － | ＋ | － | ＋ | ＋ | － | － |
| H37 | ＋ | － | ＋ | － | ＋ | － | ＋ | ＋ | － | － |
| H38 | ＋ | － | ＋ | － | ＋ | － | ＋ | ＋ | － | － |
| H39 | ＋ | － | ＋ | － | ＋ | － | ＋ | ＋ | － | － |
| H40 | ＋ | － | ＋ | － | ＋ | － | ＋ | ＋ | － | － |
| H41 | ＋ | － | ＋ | － | ＋ | － | ＋ | ＋ | － | － |
| H42 | ＋ | － | ＋ | － | ＋ | － | ＋ | ＋ | － | － |
| H43 | ＋ | － | ＋ | － | ＋ | － | ＋ | ＋ | － | － |
| H44 | ＋ | － | ＋ | － | ＋ | － | ＋ | ＋ | － | － |
| H45 | － | － | ＋ | － | ＋ | － | ＋ | － | － | － |
| H46 | ＋ | － | ＋ | － | ＋ | － | ＋ | ＋ | － | － |
| H47 | ＋ | － | ＋ | － | ＋ | － | ＋ | ＋ | － | － |
| H48 | ＋ | － | ＋ | － | ＋ | ＋ | ＋ | ＋ | － | － |
| H49 | ＋ | － | ＋ | － | ＋ | － | ＋ | ＋ | － | － |
| H50 | ＋ | － | ＋ | － | ＋ | － | ＋ | ＋ | － | － |
| H51 | － | － | ＋ | － | ＋ | － | ＋ | ＋ | － | － |
| H52 | ＋ | － | ＋ | － | ＋ | － | ＋ | ＋ | － | － |
| H53 | ＋ | － | ＋ | － | ＋ | － | ＋ | ＋ | － | － |
| H54 | ＋ | － | ＋ | － | ＋ | ＋ | ＋ | ＋ | － | － |
| H55 | ＋ | － | ＋ | － | ＋ | － | ＋ | ＋ | － | － |
| H56 | ＋ | － | ＋ | － | ＋ | － | ＋ | ＋ | － | － |
| H57 | ＋ | － | ＋ | － | ＋ | － | ＋ | － | － | － |
| H58 | ＋ | － | ＋ | － | ＋ | ＋ | ＋ | ＋ | － | － |
| H59 | ＋ | － | ＋ | － | ＋ | － | ＋ | ＋ | － | － |
| H60 | ＋ | － | ＋ | － | ＋ | － | ＋ | ＋ | － | － |
| H61 | ＋ | － | ＋ | － | ＋ | － | ＋ | ＋ | － | － |
| H62 | － | － | ＋ | － | ＋ | － | ＋ | ＋ | － | － |
| H63 | ＋ | － | ＋ | － | ＋ | － | ＋ | ＋ | － | － |
| H64 | ＋ | － | ＋ | － | ＋ | － | ＋ | ＋ | － | － |
| H65 | ＋ | － | ＋ | － | ＋ | － | ＋ | ＋ | － | － |
| H66 | ＋ | － | ＋ | － | ＋ | ＋ | ＋ | － | － | － |
| H67 | ＋ | － | ＋ | － | ＋ | － | ＋ | ＋ | － | － |
| H68  H69 | ＋  ＋ | －  － | ＋  ＋ | －  － | ＋  ＋ | －  － | ＋  ＋ | ＋  ＋ | －  － | －  － |
| H70 | ＋ | － | ＋ | － | ＋ | － | ＋ | ＋ | － | － |
| H71 | ＋ | － | ＋ | － | ＋ | － | ＋ | ＋ | － | － |
| H72 | － | － | ＋ | － | ＋ | － | ＋ | ＋ | － | － |
| H73 | ＋ | － | ＋ | － | ＋ | － | ＋ | ＋ | － | － |
| H74 | ＋ | － | ＋ | － | ＋ | ＋ | ＋ | ＋ | － | － |
| H75 | ＋ | － | ＋ | － | ＋ | － | ＋ | － | － | － |
| H76 | ＋ | － | ＋ | － | ＋ | － | ＋ | ＋ | － | － |
| H77 | ＋ | － | ＋ | － | ＋ | － | ＋ | ＋ | － | － |
| H78 | ＋ | － | ＋ | － | ＋ | － | ＋ | ＋ | － | － |

Note: HI ~ H78 is the strain number.
